# Histopathological and molecular analysis in dermis and epidermis of patients with systemic and localized scleroderma

**DOI:** 10.1186/1546-0096-12-S1-P304

**Published:** 2014-09-17

**Authors:** Betul Sozeri, Huseyin Aktug, Vildan Bozok Cetintas, Basak Yildiz, Emel Ebru Pala, Fatih Oltulu

**Affiliations:** 1 Pediatric Rheumatology, Ege University, Izmir, Turkey; 2 Histology and Embryology, Ege University Faculty Of Medicine, Izmir, Turkey; 3 Medical Biology, Ege University Faculty Of Medicine, Izmir, Turkey; 4 Pediatrics, Ege University Faculty Of Medicine, Izmir, Turkey; 5 Pathology, Tepecik Education And Research Hospital, Izmir, Turkey

## Introduction

Scleroderma is a highly complex disorder in its clinical manifestations and pathogenesis. It has a wide range of clinical manifestations due to varying degrees of vasculopathy, autoimmunity, altered endothelium function, and abnormal fibrosis which were accused in the pathogenesis of the disease.

## Objectives

The aim of this study be made of the cases diagnosed in childhood with immune histochemical analysis is to shed light on the pathogenesis of the disease.

## Methods

Single-blind clinical trial. The tissue samples obtained from patients PAS, hematoxylin and eosin, E-Cadherin, CTGF, Tunnel, and staining for TGF-β1 is applied and evaluated by light microscopy. Additionally, we have analyzed both TGFB1 level and mRNA expression analysis in plasma and tissue samples from patients. A total of 15 patients were enrolled in the study. These patients, who were chosen from, systemic (n = 8) or localized (n = 7) scleroderma patients clinically received, and for the disease of any systemic untreated and scleroderma other than skin disease.

## Results

In the study a total of 22 tissue samples (15 diseased tissue, healthy tissue and 7) were used. The mean age of onset was 9.2 ± 1.2 years and the mean age of diagnosis was 15.3 ± 3.2 years. All patients with systemic sclerosis have antinuclear antibody (ANA) titer 1/160-1/640. ANA positivity in patients with localized scleroderma were no. Clinically with different characteristics in the two subgroups of the histopathology examination was shown to be distinct from the tissue level.

## Conclusion

Playing a fundamental role in the pathogenesis of the TGF-B levels in both plasma and skin have been shown to be locally high. Particularly in patients with systemic scleroderma this elevation was found to be more pronounced. Treatment for this disease is still questionable results obtained will throw light on new treatment possibilities.

## Disclosure of interest

None declared.

